# Phenotypic and Molecular Traits of *Staphylococcus coagulans* Associated with Canine Skin Infections in Portugal

**DOI:** 10.3390/antibiotics10050518

**Published:** 2021-05-02

**Authors:** Sofia Santos Costa, Valéria Oliveira, Maria Serrano, Constança Pomba, Isabel Couto

**Affiliations:** 1 Global Health and Tropical Medicine (GHTM), Instituto de Higiene e Medicina Tropical (IHMT), Universidade Nova de Lisboa (UNL), Rua da Junqueira 100, 1349-008 Lisboa, Portugal; mmm0005@ihmt.unl.pt (V.O.); a21000870@ihmt.unl.pt (M.S.); 2 Centre of Interdisciplinary Research in Animal Health (CIISA), Faculty of Veterinary Medicine, University of Lisbon, Avenida da Universidade Técnica, 1300-477 Lisboa, Portugal; cpomba@fmv.ulisboa.pt; 3 GeneVet, Laboratório de Diagnóstico Molecular Veterinário, Rua Quinta da Nora Loja 3B, 2790-140 Carnaxide, Portugal

**Keywords:** *Staphylococcus coagulans*, *Staphylococcus schleiferi*, skin infections, dogs, antibiotic resistance, methicillin resistance, wild-type populations, genetic lineages, plasmids

## Abstract

*Staphylococcus coagulans* is among the three most frequent pathogens of canine pyoderma. Yet, studies on this species are scarce. Twenty-seven *S. coagulans* and one *S. schleiferi*, corresponding to all pyoderma-related isolations from these two species at two veterinary laboratories in Lisbon, Portugal, between 1999 and 2018 (Lab 1) or 2018 (Lab 2), were analyzed. Isolates were identified by the analysis of the *nuc* gene and urease production. Antibiotic susceptibility towards 27 antibiotics was evaluated by disk diffusion. Fourteen antibiotic resistance genes were screened by PCR. Isolates were typed by *Sma*I-PFGE. Two *S. coagulans* isolates (2/27, 7.4%) were methicillin-resistant (MRSC, *mecA*+) and four (4/27, 14.8%) displayed a multidrug-resistant (MDR) phenotype. We observed resistance to penicillin (17/27, 63.0%), fluoroquinolones (11/27, 40.7%), erythromycin and clindamycin (3/27, 11.1%), fusidic acid (3/27, 11.1%) and tetracycline (1/27, 3.7%). The *blaZ* and *erm*(B) genes were carried by 16 and 1 isolates resistant to penicillin and erythromycin/clindamycin, respectively. Only three *S. coagulans* carried plasmids. The single *S. schleiferi* isolate presented an MDR phenotype. *Sma*I-PFGE revealed a limited genetic diversity of *S. coagulans*, with a predominant lineage present from 2001 to 2018. This study describes the first MRSC causing canine infection in Portugal and reveals a high burden of antimicrobial resistance, with the emergence of MDR phenotypes within the main lineages.

## 1. Introduction

Pyoderma is a common skin infection in dogs and the main cause for antimicrobial use in small animal veterinary medicine [1]. Coagulase-positive staphylococci are the main pathogenic agents of canine pyoderma; *Staphylococcus pseudintermedius* accounts for up to 90% of pyoderma-related staphylococcal infections, followed by *Staphylococcus coagulans* (previously known as *Staphylococcus schleiferi* subsp. *coagulans*) and *Staphylococcus aureus* [1,2,3]. Coagulase-negative staphylococci, like *Staphylococcus epidermidis*, are rare agents of canine pyoderma, frequently in association with *S. pseudintermedius* [1].

The species *S. schleiferi* was first described in 1988 by Freney and colleagues in human clinical isolates [4], and later reported as a member of the human axilla microflora [5,6]. In 1990, Igimi and colleagues identified several isolates of *S. schleiferi* from dogs with otitis externa that presented distinct characteristics from the *S. schleiferi* isolated from humans, leading to the proposal of a new subspecies denominated *S. schleiferi* subsp. *coagulans* [7]. *S. schleiferi* subsp. *coagulans* differed from *S. schleiferi* subsp. *schleiferi*, among other traits, by the production of the enzymes coagulase and urease [7]. In 2020, a phylogenomic analysis of the *Staphylococcus* genus led to the re-classification of *S. schleiferi* subsp. *coagulans* as the new species *S. coagulans* and of *S. schleiferi* subsp. *schleiferi* as *S. schleiferi* [8].

*S. coagulans* is part of the dog skin microflora; it has been isolated from the skin [9] and ear canal of healthy dogs [10], although its presence may be registered in low numbers [11]. This species has also been isolated from the skin of healthy cats [12] and, more recently, from mouth samples of healthy seals [13]. *S. coagulans* is an opportunistic pathogen. The first case of skin infection caused by *S. coagulans* was reported by Bes and colleagues in 2002 [14]. Since then, this species has been associated with skin, ear or urinary infections in dogs, many of which are recurrent [10,15,16,17,18,19]. Albeit rare, there have been reports of human opportunistic infections caused by *S. coagulans* [20,21,22,23,24], some of which are potentially linked to cases of dog-to-human transmission of *S. coagulans* strains [22,23,24].

Antimicrobial resistance is a frequent trait of staphylococci and increasing rates of antimicrobial resistance have been documented in major animal staphylococcal pathogens, such as *S. pseudintermedius* and *S. aureus* [25]. For *S. coagulans*, there are a limited number of reports on the rate of antimicrobial resistance worldwide. The first methicillin-resistant *S. coagulans* (MRSC) isolates were identified in the US in 2003 [15]. These MRSC isolates, resistant to all beta-lactam antibiotics except fifth-generation cephalosporins, were linked to recurrent cases of canine pyoderma [15]. Since then, several US-based studies have indicated frequency rates of MRSC from 0% [26] up to 75%, mainly associated with cases of recurrent skin or ear infections in dogs, many of which subjected to previous antibiotic therapy [15,27,28,29,30,31,32,33]. In fact, previous treatment with penicillin or a cephalosporin has been reported as a risk factor for the emergence of MRSC [34]. Studies conducted from other geographic regions have also shown a variability in frequency rates of MRSC isolates linked to skin, ear or urinary infections, from 0% in Slovenia [35], the UK [16], Scotland [36], Italy [37] Portugal [38], Australia [39] and Japan [10] to rates of 12.5–20% in Brazil [17,40], 24% in Korea [41], ca. 30% in Japan [18,42] and 70% in Thailand [43]. However, the MRSC frequency rates determined for some of these studies may not be precise, due to the low number of isolates studied, lack of differentiation between *S. schleiferi* subspecies and, most importantly, absence of clinical breakpoints for the detection of methicillin resistance in these species, which were only recommended by CLSI from 2018 onwards [44]. Resistance to other antibiotic classes has also been reported, associated or not to methicillin-resistance, and usually includes resistance to fluoroquinolones [37,45], penicillin, erythromycin, clindamycin, gentamicin, chloramphenicol, tetracycline and fusidic acid [10,18,19,29,30,32,34,39,43]. Despite the low prevalence of *S. coagulans*, and consequently, of the absolute numbers of strains analyzed, an increasing trend of antimicrobial resistance has been reported for this species [33,46], which may affect the management of infections caused by *S. coagulans*.

In a previous study conducted in Portugal, *S. coagulans* and *S. schleiferi* were associated with over 11% of skin, ear and urinary infections in pets during a 16-year period (1999–2014) in a veterinary research laboratory receiving samples from a veterinary teaching hospital and private practices in Lisbon area [38]. Although no MRSC isolates were detected in that earlier study, an increasing trend of antimicrobial resistance burden was observed for *S. coagulans* during the 16-year timeframe [38]. In the present work, we expand that previous study, focusing on all the *S. coagulans* and *S. schleiferi* associated with skin infections, isolated from 1999 to 2018, as well as all *S. coagulans* and *S. schleiferi* related to skin infections and collected during 2018 at a second private veterinary diagnostic laboratory. The aims of our study were to analyze the antimicrobial resistance burden of these pathogens and their association with genotypic determinants of resistance, plasmid content and molecular lineages; the latter two traits have not been studied yet in Portugal and have been rarely documented worldwide.

## 2. Results

### 2.1. Identification of S. coagulans and S. schleiferi Isolates

The identification of *S. coagulans* and *S. schleiferi* isolates among an initial collection of 89 pyoderma-related *Staphylococcus* isolates was performed or confirmed by the species-specific *nuc*-PCR strategy proposed by Sasaki and colleagues [47], followed by detection of urease activity [44]. Of the 89 isolates screened, 28 were identified as *S. coagulans* or *S. schleiferi* by the *nuc*-PCR. Of these, 27 were urease-positive and thus identified as *S. coagulans*, whereas one isolate was urease-negative and identified as *S. schleiferi*. All isolates were collected from dogs and linked to pyoderma; two *S. coagulans* isolates were collected from the same dog, corresponding to skin swabs sampled one year apart. In sum, the study collection comprised 27 *S. coagulans* isolates; 21 collected over 19 years in a veterinary research lab (Lab 1), 7 collected during 2018 at a veterinary diagnostic lab (Lab 2) and a single *S. schleiferi* isolate collected at Lab 1 in 2016.

### 2.2. Antimicrobial Resistance Profiles and Association with Resistance Determinants

**Figure 1:**
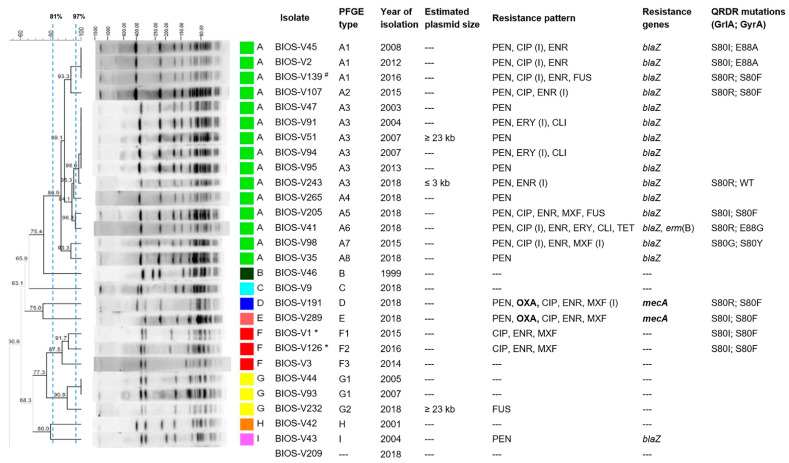
*Sma*I-PFGE macrorestriction profile analysis of the 27 *S. coagulans* and 1 *S. schleiferi* isolates associated with canine skin infections and their correlation with plasmid profiles, as well as their phenotypic and genotypic resistance traits. The symbol (#) indicates the *S. schleiferi* isolate. The symbol (*) highlights the two *S. coagulans* isolates collected from the same dog. The dendrogram was built using Bionumerics and the UPGMA algorithm, using the Dice coefficient, and an optimization of 0.5% and tolerance of band of 1%. The dashed lines correspond to the similarity criteria for considering isolates belonging to the same PFGE type (≥81%) or subtype (≥97%). PFGE: pulsed-field gel electrophoresis; PEN: penicillin; CIP: ciprofloxacin; ENR: enrofloxacin; MXF: moxifloxacin; ERY: erythromycin; CLI: clindamycin; FUS: fusidic acid; TET: tetracycline; (I) intermediate phenotype; QRDR: quinolone-resistance determining region; S: serine; I: isoleucine; E: glutamic acid; A: alanine; R: arginine; F: phenylalanine; G: glycine; Y: tyrosine; WT: wild-type.

The antibiotic susceptibility profiles of all *S. coagulans* isolates are discriminated in Table 1 and Figure 1. Inhibition zone diameters for all antibiotics studied are shown in Tables S1–S3 of the Supplementary Materials.

Antibiotic susceptibility testing showed that of the 27 isolates in study, 16 (16/27, 59.3%) were resistant to one or two antibiotics of distinct classes and 4 (4/27, 14.8%) displayed a multidrug resistance phenotype, showing resistance to, at least, three different classes of antibiotics. Only seven (7/27, 25.9%) were susceptible to all antibiotics tested. Two *S. coagulans* isolates (2/27, 7.4%) were resistant to oxacillin and carried the *mecA* gene, being classified as MRSC, and thus, resistant to all beta-lactams, except for fifth-generation cephalosporins. The *mecA* gene was not detected in the remaining isolates.

The most common resistance pattern for *S. coagulans* was monoresistance to penicillin, observed in six isolates (6/27, 22.2%), followed by resistance to penicillin and fluoroquinolones (5/27, 18.5%). The MDR isolates were characterized by resistance to penicillin, fluoroquinolones and fusidic acid (1 out of 4 isolates), or penicillin, erythromycin and clindamycin (2 out of 4 isolates), and additional resistance to fluoroquinolones and tetracycline (1 out of 4 isolates). The two MRSC isolates also showed resistance to fluoroquinolones.

Resistance to penicillin was the most frequently observed phenotype, detected in 17 *S. coagulans* (17/27, 63.0%). Of these, one isolate presented a zone inhibition diameter (ZD) of 31 mm but showed a sharp inhibition border, being considered a beta-lactamase producer and penicillin-resistant. Another isolate showed an ZD of 33 mm and a fuzzy border but carried the *mecA*, and thus, was also considered resistant to penicillin [48]. The *blaZ* gene encoding the beta-lactamase BlaZ was present in 16 of the 17 penicillin-resistant isolates.

Resistance to fluoroquinolones was observed in 11 out of the 27 *S. coagulans* isolates (11/27, 40.7%). These 11 isolates were resistant (*n* = 9) or intermediate (*n* = 2) to enrofloxacin; 10 (37.0%) were resistant (*n* = 6) or intermediate (*n* = 4) to ciprofloxacin; and 6 (22.2%) were resistant (*n* = 4) or intermediate (*n* = 2) to moxifloxacin. Sequencing the quinolone-resistance determining region (QRDR) of the *grlA* and *gyrA* genes of the 11 isolates presenting fluoroquinolone resistance revealed six distinct patterns of mutations in GrlA and GyrA (Figure 1). The pair GrlA: S80I/GyrA:S80F, observed in four isolates, was the only associated with a resistance phenotype to the three fluoroquinolones tested. On the other hand, the single GrlA mutation S80R was present in one isolate with intermediate phenotype only towards enrofloxacin. The remaining mutation patterns GrlA:S80I/GyrA:E88A, GrlA:S80R/GyrA:S80F, GlA:S80R/GyrA:E88G and GrlA:S80G/GyrA:S80Y were all linked to resistance or intermediate to either ciprofloxacin or enrofloxacin.

Resistance to erythromycin was detected in three *S. coagulans* isolates (3/27, 11.1%), two of which were intermediate. These three isolates showed also constitutive resistance to clindamycin (3/27, 11.1%). From the seven macrolide-lincosamide resistance genes screened, only *erm*(B) was detected in the single isolate classified as resistant to both erythromycin and clindamycin. No resistance gene was detected for the remaining two isolates resistant to clindamycin and intermediate to erythromycin.

Resistance to fusidic acid was detected in two *S. coagulans* isolates (2/27, 7.4%) and to tetracycline in a single isolate (1/27, 3.7%), but none of these isolates harbored any of the resistance determinants screened, *fusB*/*fusC* or *tetK*/*tetM/tetL*, respectively.

The single *S. schleiferi* isolate of this collection presented an MDR phenotype, with resistance to penicillin, fluoroquinolones and fusidic acid, and harbored the *blaZ* gene and the QRDR mutations GrlA:S80R and GyrA:S80F. Neither of the fusidic acid resistance genes screened, *fusB*/*fusC*, were detected in this isolate.

None of the *S. coagulans* or *S. schleiferi* isolates were resistant to chloramphenicol, quinupristin-dalfopristin, trimethoprim-sulfamethoxazole, linezolid, rifampicin, minocycline, tigecycline nor to the aminoglycosides gentamicin, tobramycin, kanamycin or amikacin.

### 2.3. Distributions of Zone Inhibition Diameters for Antibiotics with No Breakpoints Established

**Figure 2:**
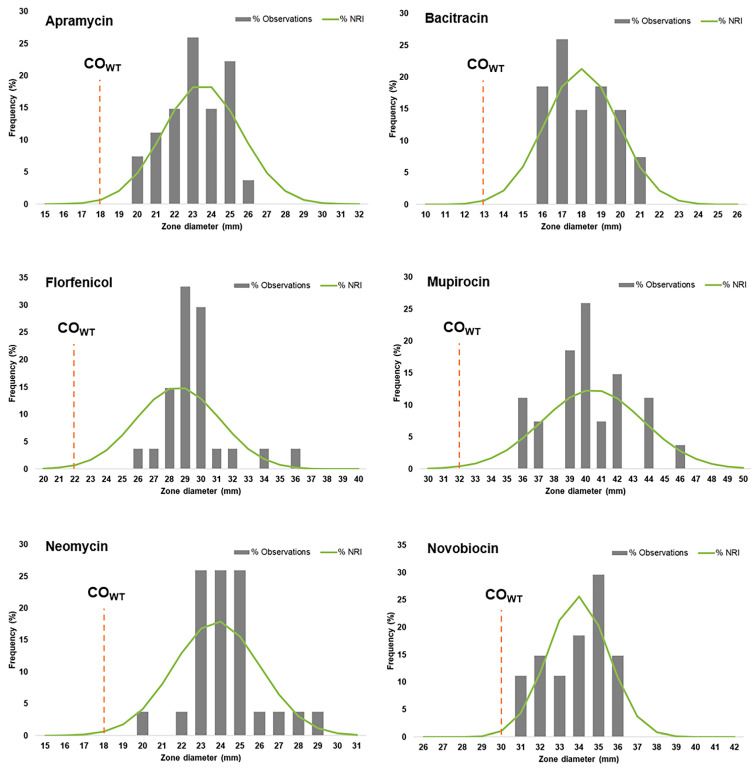
Distributions of inhibition zone diameters for six antibiotics with no established breakpoints for *S. coagulans* and corresponding cut-off value (CO_WT_). The CO_WT_ value (dashed orange lines) was calculated using the Normalized Resistance Interpretation (NRI) method. Grey columns represent the distribution of zone diameters; the green line indicates the NRI-generated normalized distribution of the putative WT populations.

One of the aims of this study was to evaluate the overall burden of antimicrobial resistance in *S. coagulans* to a wide set of antibiotics for multiple classes, independently of their recommended use for canine skin infections [1,2]. This evaluation is impaired by the limited number of breakpoints recommended for *S. coagulans*/*S. schleiferi* by Clinical Laboratory and Standards Institute (CLSI) or European Committee on Antimicrobial Susceptibility Testing (EUCAST). For most antibiotics, the recommendation is to use breakpoints established for *Staphylococcus* spp. isolated from humans [48] and/or established for *S. aureus* [50]. To overcome this limitation, we estimated the ZD-based cut-off value (CO_WT_) of *S. coagulans* for antibiotics with no breakpoints available. This parameter corresponds to the smallest ZD value of the wild-type population (the population without phenotypically expressed resistance mechanisms) [51]. It also allows the detection of putative non-wild type populations (with phenotypically expressed resistance mechanisms) in a given bacterial collection. Thus, the distributions of zone inhibition diameters were analyzed to evaluate the possible presence of resistance determinants in *S. coagulans* for the antibiotics mupirocin, florfenicol, neomycin, bacitracin, novobiocin and apramycin (Figure 2). This analysis only took into consideration the 27 isolates identified as *S. coagulans*.

The Normalized Resistance Interpretation (NRI) method was used to estimate the distribution of the putative wild-type (WT) population and calculate the CO_WT_ value for this population. All zone diameter distributions were unimodal. The normalized distributions of the WT populations were validated as they all included over 15 observations and each associated standard deviation (SD) was below the acceptable SD upper limit of 3.38 mm (Table 2). The application of the estimated CO_WT_ values showed the absence of NWT populations of *S. coagulans* towards all the six antibiotics tested, indicating the absence of isolates with resistance mechanisms to these antibiotics in our collection.

### 2.4. Plasmid Profiling and Association with Antibiotic Resistance

Plasmid DNA was isolated for all isolates. From the 27 *S. coagulans* studied, only 3 (3/27, 11.1%) carried plasmids. Two isolates showed a single plasmid of ≥23 kb, whereas the remaining *S. coagulans* presented a single plasmid of ≤3 kb. Of the two isolates harboring a large plasmid, one showed resistance to penicillin and carried the *blaZ* gene, while the other was only resistant to fusidic acid with no associated resistance determinant detected. The isolate harboring the small plasmid showed resistance to penicillin and the *blaZ* gene as well as an intermediate phenotype towards enrofloxacin.

### 2.5. Molecular Typing and Association with Antimicrobial Resistance Phenotypes

All isolates, but one, were subjected to molecular typing by *Sma*I-PFGE (Figure 1). The collection comprised nine pulsed-field gel electrophoresis (PFGE) types, classified A to I, and thirteen subtypes. The PFGE type A was the most common, represented by over half of the *S. coagulans* isolates (14/27, 51.8%) and comprising eight subtypes. PFGE types F and G each included three isolates (3/27, 11.1%) and comprised three and two subtypes, respectively. The remaining six PFGE types were represented by a single isolate. The single *S. schleiferi* isolate presented a PFGE pattern indistinguishable from *S. coagulans*, sharing the PFGE type A1 together with two *S. coagulans* isolates. The two *S. coagulans* isolates collected one year apart from the same dog belonged to different subtypes of PFGE type F. A calculation of the Simpson’s Index of Diversity (SID) based on the *S. coagulans* PFGE types revealed that this collection is not very diverse (SID = 0.70, CI: 0.51–0.89).

The main PFGE type A was present in isolates collected from 2001 to 2018. All isolates with this PFGE pattern showed resistance to at least one antibiotic. Additionally, all four MDR isolates belonged to PFGE type A. The two MRSC isolates belonged to two distinct and unique PFGE types (D and E). We observed an increase in antimicrobial resistance accompanying the year of isolation of the *S. coagulans* isolates, detectable within the main PFGE types. For example, up to 2014, isolates from PFGE type A (*n* = 7) presented monoresistance to penicillin (*n* = 3) with additional resistance to fluoroquinolones (*n* = 2) or macrolides-lincosamides (*n* = 2, MDR). However, from 2015 to 2018, the remaining isolates with PFGE type A (*n* = 7) presented more diverse and additional resistance phenotypes, namely resistance to penicillin-fluoroquinolones-fusidic acid (*n* = 1, MDR) or resistance to penicillin-fluoroquinolones-macrolides-lincosamides-tetracycline (*n* = 1, MDR), besides the patterns previously detected (monoresistance to penicillin, *n* = 2 or resistance to penicillin-fluoroquinolones, *n* = 3). Within PFGE type F, the emergence of resistance to fluoroquinolones was detectable in the two isolates collected in 2015/2016, whereas for PFGE type G, resistance to fusidic acid was detected in the 2018 isolate.

The increasing antimicrobial resistance was also illustrated by the detection of the two MRSC isolates in 2018, belonging to PFGE types D and E.

## 3. Discussion

Despite being a relevant agent of canine pyoderma, *S. coagulans* is still poorly characterized regarding antimicrobial resistance traits and, particularly, its circulating clonal lineages. A previous study in Portugal surveyed the antimicrobial resistance burden of *S. coagulans* associated with several types of infection during a 16-year period (1999–2014). During that timeframe, no MRSC isolates were detected, but MDR phenotypes were observed [38]. In this work, we expanded the collection to encompass the years 2015 up to 2018; additionally, we included all isolates collected during 2018 at a second veterinary diagnostic laboratory. We also broadened the analysis to plasmid profiling and molecular typing. However, we focused our study on isolates related to skin infections.

This study revealed that a significant proportion of all the non-*S. pseudintermedius* or non-*S. aureus* isolates collected from companion animals skin infections during the time period studied was identified as *S. coagulans* (27/89, 30.3%), while a single isolate (1/89, 1.1%) corresponded to *S. schleiferi*. The low total number of *S. coagulans* or *S. schleiferi* isolates collected during this 19-year period (1999–2018) reflects the lower rate of canine skin infections caused by these pathogens when compared to *S. pseudintermedius* [3]. Several studies have shown that *S. coagulans* is commonly the second most isolated staphylococci isolated from skin samples of canine pyoderma, in frequencies varying from 3% [32,52] to up to 10% [18]. The predominance of *S. coagulans* over *S. schleiferi* in canine skin samples observed in our study agrees with other studies [31,32], although some works documented similar or higher frequency of *S. schleiferi* in canine pyoderma [53,54].

We registered a high rate of antimicrobial resistance in the collection studied. In total, 75% of the isolates (21/28, considering *S. coagulans* and *S. schleiferi*) were resistant to at least one antibiotic and 17.9% (5/28) were MDR. This study also reports the first MRSC isolates in dogs in Portugal. A comparison of the antimicrobial resistance in the *S. coagulans* isolated between the 1999–2014 period (13 isolates obtained during the study by Couto and colleagues [38]) with the isolates collected afterward (2015–2018, *n* = 14) revealed an increase in the antimicrobial-resistant isolates. This was manifested by the decrease in the total number of fully-susceptible isolates (*n* = 5 in 1999–2014 to *n* = 2 in 2015–2018) and the increase in total number of isolates resistant to one or two classes of antibiotics (*n* = 6 in 1999–2014 to *n* = 10 in 2015–2018). Most importantly, we reported the emergence of two MRSC isolates from distinct lineages (different PFGE types) in the last year surveyed (2018). We also describe the emergence, since 2016, of resistance to fusidic acid, which is widely used in the topical treatment of human and canine skin infection caused by either *S. coagulans* or *S. schleiferi*. Worryingly, this increase in antimicrobial resistance in *S. coagulans* has also been reported in other countries, namely in the US [32,33,55]. The frequency rates of MRSC and MDR detected in our study are higher than the ones previously reported for other European countries [16,35,36,37], although there is a lack of recent surveys for direct comparison purposes.

The two MRSC isolates described in this study were also resistant to fluoroquinolones, as observed in other studies [30]. Nearly half of the *S. coagulans* studied were resistant to fluoroquinolones, and all carried QRDR mutations in a diverse range of combinations. High levels of resistance to fluoroquinolones have been reported worldwide in *S. coagulans* collected from dogs, irrespective of methicillin-resistance status, including in the UK [16], Italy [37], Japan [18] and the US [19]. The description of QRDR mutations in *S. coagulans* is sparce. A study by Intorre and colleagues identified the mutations GrlA: S80R or GyrA: E88G in *S. coagulans* resistant to old generation fluoroquinolones (enrofloxacin, ciprofloxacin and orbifloxacin) yet susceptible to moxifloxacin, gatifloxacin and trovafloxacin [45]. In the present study, we identified a wider set of QRDR mutations. Among the six different patterns of mutations identified, only the combination GrlA:S80I and GyrA:S80F was linked to resistance to the three fluoroquinolones tested, whereas a single mutation in GrlA (S80R) was associated with an intermediate phenotype towards enrofloxacin.

In the last decades, a decrease has been observed in the susceptibility to penicillin, erythromycin, clindamycin and to a lesser extent gentamicin or other aminoglycosides and tetracycline in *S. coagulans*, but the resistance determinants associated with these resistance phenotypes is scarcely documented [10,18,19,36,41,42,43]. As observed in other studies, resistance to penicillin was linked to the presence of the *blaZ* gene, encoding the beta-lactamase BlaZ, except for one MRSC isolate which did not carry this determinant. Contrary to other staphylococci, *blaZ* carriage was not associated with large multiresistance plasmids. Resistance to macrolides and lincosamides is common in *S. coagulans* and has been linked to the presence of *erm*(B) and/or *erm*(C) genes [41,42,43]. In our collection, only one out of the three isolates resistant to these antibiotics harbored the *erm*(B). Although negative for the other six resistance genes screened [*erm*(A), *erm*(C), *msrA*, *mph*(C), *vga*(A) and *vga*(C)], the remaining two resistant isolates could carry other determinants, such as additional *erm* genes like *erm*(43) or *lnu*(B), which are not usually linked to plasmids [56]. Resistance to fusidic acid was detected in three *S. coagulans* and *S. schleiferi* collected from 2016 onwards, highlighting the recent emergence of resistance to this antibiotic. However, none carried the two resistance determinants screened, *fusB*/*fusC*, which are usually associated with plasmids or other mobile genetic elements. Resistance to this antibiotic could be linked to mutations occurring in the *fusA* gene, which are common in other staphylococcal species [56]. Regarding resistance to tetracycline, none of the *tet* genes screened was found in the single tetracycline-resistant *S. coagulans* detected. However, this isolate could carry the *tet*(O) determinant, as observed in the study by Chanchaithong and colleagues [43].

Despite the increase registered in antimicrobial resistance in *S. coagulans* in Portugal in the last two decades, resistance phenotypes are still mainly restricted to a few antibiotic classes, namely beta-lactams, fluoroquinolones, macrolide-lincosamides and fusidic acid. Reports from other countries such as Thailand [43] showed a higher diversity of resistance phenotypes and resistance determinants in *S. coagulans*. The low diversity of resistance genes encountered in our collection could be associated with the low abundance of plasmids.

Our knowledge on the molecular epidemiology of *S. coagulans* is rather limited to a few studies with a limited number of isolates. These have used *Sma*I-PFGE to evaluate the clonality of this species, indicating a high clonality within the species and that *S. coagulans* and *S. schleiferi* often present indistinguishable PFGE patterns [10,19,29,33,43]. Our data also show that *S. coagulans* collected in Portugal (Lisbon area) throughout the last two decades present a low genetic diversity (Simpson’s index of diversity, SID = 0.70), with a predominant lineage encompassing over half of the isolates collected through almost the entire time span of the collection. Nevertheless, some heterogeneity was found within this predominant lineage, with isolates varying in resistance phenotypes and resistance determinants.

We also analyzed the ZD distributions and corresponding CO_WT_ values for six antibiotics (apramycin, bacitracin, florfenicol, mupirocin, neomycin and novobiocin) with no breakpoints available for *S. coagulans*. These antibiotics are not used or rarely recommended (mupirocin) for the treatment of skin infections in dogs [1,2]. Nevertheless, the emergence and dissemination of resistance to these antibiotics in several staphylococci of animal origin is worrisome [56]. Thus, the availability of cut-off values that allow the identification of isolates potentially harboring resistance mechanisms to these antibiotics could be a valuable tool in future antimicrobial resistance surveillance studies in *S. coagulans*.

The results gathered in this study are particularly worrisome when analyzed in conjunction with data on the classes of antibiotics most prescribed in small animal practice. Data from the European Medicines Agency (EMA) and a report by Oliveira and colleagues highlight that beta-lactams, fluoroquinolones, macrolides and tetracyclines represent the majority of antibiotics prescribed for treatment of skin infections in small animals [57,58]. Although fusidic acid is not listed in these two studies, it is recommended for the topical management of canine pyoderma [1,2] and, worryingly, is frequently used (for human or animal use) without requiring medical prescription. Our data suggest that the high burden of antimicrobial resistance in *S. coagulans* may reflect the usage of antibiotics recommended for the treatment of canine pyoderma.

## 4. Materials and Methods

### 4.1. Bacterial Isolates

The initial collection studied (total *n* = 89) included all isolates previously identified as *S. schleiferi* or *Staphylococcus* spp. (non-*S. pseudintermedius* and non-*S. aureus*) causing skin infections in pets collected over 19 years (1999–2018) at a veterinary research laboratory providing diagnostic services for a veterinary teaching hospital and private veterinary clinics in the Lisbon area (Lab 1, *n* = 40) and at a private veterinary diagnostic clinic during 2018 (Lab 2, *n* = 49) (Table 3). This collection included thirteen isolates collected from 1999 to 2014 at Lab 1 and previously identified as *S. schleiferi* that were partially characterized in a previous study [38]. The type strains *S. coagulans* DSM 6628^T^ and *S. schleiferi* DSM 4807^T^ were acquired from DSMZ GmbH (Germany) and included in the study as controls. All isolates were grown in tryptone soya broth or agar (TSB/TSA, Thermo Scientific™ Oxoid™, Basingstoke, UK) at 37 °C, at 180 rpm (for broth cultures). Bacterial stocks were kept at −80 °C in TSB supplemented with 10% (*v*/*v*) glycerol.

### 4.2. Total DNA and Plasmid DNA Isolation

Total DNA was isolated by the boiling method [59] and used as a template for all PCR protocols. Plasmid DNA was isolated with NZYMiniprep Kit (NZYTech, Lisbon, Portugal), according to the manufacturer’s recommendations and with the addition of an incubation step with lysostaphin at 35 mg/L for 60 min at 37 °C following resuspension of the bacterial pellet with buffer A1. All plasmid preparations were analyzed in 1% agarose gel electrophoresis in TAE 1X buffer at 80 V for 90 min using the molecular ladder lambda/*Hind*III (Thermo Scientific™).

### 4.3. Identification of the Isolates

The identification of the isolates as *S. coagulans* or *S. schleiferi* was confirmed by a species-specific *nuc* PCR approach described by Sasaki et al. [47], after decreasing the annealing temperature from 56 °C to 50 °C, to obtain a good specific amplification for the two type strains used as positive controls. Total DNA from the type or reference strains *S. pseudintermedius* DSM 21284^T^ (DSMZ GmbH), *S. aureus* ATCC^®^ 259323™ and *S. epidermidis* ATCC^®^ 12228™ (LGC Standards, S.L.U, Barcelona, Spain) were included as negative controls. The differentiation between *S. coagulans* and *S. schleiferi* was carried out, for each isolate, by detection of urease activity after growth in Christensen urea agar at 35 °C for up to 48 h [44]. Conversion of the medium color from yellow to pink indicates the presence of urease activity. The urease-negative strains *S. schleiferi* DSM 4807^T^ and *Escherichia coli* ATCC^®^ 2922™, and the urease-positive strains *S. coagulans* DSM 6628^T^ and *Klebsiella pneumoniae* ATCC^®^ BAA 1706™ were included as controls.

### 4.4. Antibiotic Susceptibility Testing

Antibiotic susceptibility was determined by agar disk diffusion on Mueller-Hinton agar plates (Thermo Scientific™ Oxoid™) according to CLSI recommendations for bacteria isolated from animals [48] or EUCAST recommendations [50]. We studied a wide panel of antibiotics, not only used in veterinary practice, but also human medicine, to evaluate the overall burden of antimicrobial resistance in *S. coagulans*. Antibiotic disks were acquired from Thermo Scientific™ Oxoid™ or MAST Group (pradofloxacin) (Liverpool, UK). Susceptibility profiles were interpreted according to CLSI VET01S-ED5 recommendations for enrofloxacin (5 μg), clindamycin (2 μg) and tetracycline (30 μg) [48] or according to CLSI M100-S30 (bacteria isolated from humans) for penicillin (10 U), oxacillin (1 μg), ciprofloxacin (5 μg), moxifloxacin (5 μg), gentamicin (10 μg), erythromycin (15 μg), minocycline (30 μg), trimethoprim/sulfamethoxazole (1.25/23.75 μg), chloramphenicol (30 μg), linezolid (30 μg), rifampicin (5 μg) [49]; or according to EUCAST for tobramycin (10 μg), kanamycin (30 μg), amikacin (30 μg), quinupristin-dalfopristin (15 μg), fusidic acid (10 μg) and tigecycline (15 μg) [50]. Other antibiotics were tested for which no breakpoints are established, namely pradofloxacin (5 μg), neomycin (30 μg), apramycin (15 μg), florfenicol (30 μg), novobiocin (30 μg), mupirocin (200 μg) and bacitracin (10 U). Isolates categorized as intermediate using the CLSI breakpoints were considered resistant, according to these recommendations. The D-zone test was performed for detection of inducible clindamycin resistance [49]. The reference strains *S. aureus* ATCC^®^25923™ and *S. aureus* ATCC^®^29213™ and the type strains *S. coagulans* DSM 6628^T^ and *S. schleiferi* DSM 4807^T^ were used as quality controls. Multidrug resistance (MDR) was considered as not-susceptibility to, at least, one agent of, at least, three classes of antimicrobials [60].

### 4.5. Calculation of Cut-Off (CO_WT_) Values

The ZD-based CO_WT_ values were estimated using the Normalized Resistance Interpretation (NRI) method [51,61]. This method uses the distributions of inhibition zone diameters to make a least-square regression analysis to determine the putative wild-type (WT) population, the mean inhibition zone diameter and the associated standard deviation (SD) for each species-antimicrobial agent combination. The CO_WT_ corresponds to the smallest inhibition zone diameter presented by the putative WT population and is calculated at 2.5X the SD above the mean value and rounded up to the lowest absolute value [51,61]. Thus, the CO_WT_ allows the distinction between putative WT populations (devoid of phenotypically-detectable acquired resistance mechanisms) and non-wild-type populations (NWT, with phenotypically-detectable acquired resistance mechanisms) [62]. The CO_WT_ estimated by the NRI method will include 99.4% of the WT population [61]. The NRI method was used with permission from the patent holder, Bioscand AB, TÄBY, Sweden (European patent No 1383913, US Patent No. 7,465,559). The automatic and manual excel programs were made available through courtesy by P. Smith, W. Finnegan and G. Kronvall at http://www.bioscand.se/nri/ (accessed on 2 December 2020).

### 4.6. Screening of Resistance Determinants

Carriage of the beta-lactam resistance genes *blaZ* and *mecA* was screened by PCR for all isolates, whereas the genes *erm*(A), *erm*(B), *erm*(C), *msrA*, *mph*(C), *vga*(A) and *vga*(C) for resistance to macrolides and/or lincosamides, genes *tet*(K), *tet*(L), *tet*(M) for resistance to tetracyclines and genes *fusB* and *fusC* for resistance to fusidic acid were only screened by PCR for isolates a resistant or intermediate phenotype. The presence of mutations associated with fluroquinolone resistance was screened by sequencing of the quinolone-resistance determining region (QRDR) of the genes *grlA* and *gyrA*. Amplification products were purified using the kit NZYGelpure (NZYTech, Lisboa, Portugal) and sequenced. Sequences were analyzed using the programs SnapGene Viewer (GSL Biotech; available at snapgene.com), translated to polypeptide sequnces, aligned in MEGA v. 7.0.26 and compared against the GrlA and GyrA sequences of the type strain *S. coagulans* DSM6628^T^ (GenBank assembly accession no. GCA_002901995.1). All primers used for the detection of resistance genes or mutations are listed in Supplementary Table S4, while the strains used as controls for PCR assays are discriminated in Supplementary Table S5.

### 4.7. Molecular Typing by SmaI-PFGE

All isolates in study were subjected to *Sma*I-PFGE typing. Briefly, agarose disks containing genomic DNA were prepared and restricted with *Sma*I, as previously described for *S. aureus* [63]. Restriction fragments were then resolved by PFGE in a contour-clamped homogeneous electric field (CHEF) apparatus (CHEF-DRIII, Bio-Rad, Hercules, CA, USA), using the following running parameters: 14 °C, 200 V (6 V/cm), 120°, 5 s of initial switch, 40 s of final switch and 21 h of running time [19]. In each run, control agarose disks were included containing the genomic DNA of the reference strain *S. aureus* NCTC8325 (used for gel normalization) and the PFG DNA Ladder (New England Biolabs). Macrorestriction profiles were analyzed with BioNumerics software version 7.6 (Applied Maths, Kortrijk, Belgium) using the unweighted pair group method using arithmetic averages and the Dice similarities coefficient. Band position tolerance and optimization were set at 1% and 0.5%, respectively. Isolates with a similarity coefficient ≥ 81% were considered as belonging to the same PFGE type, whereas isolates with a similarity coefficient ≥ 97% were considered as belonging to the same PFGE sub-type [64]. The genetic diversity of the collection was calculated, based on PFGE types, by Simpson’s index of diversity with a confidence interval of 95% [65].

## 5. Conclusions

This study on *S. coagulans* associated with canine pyoderma highlights a high burden of antimicrobial resistance in *S. coagulans* in Portugal during the last two decades, including the emergence of MRSC. This work also reveals a low genetic diversity and a low content of plasmids in the circulating *S. coagulans*. Yet, new lineages with relevant resistance phenotypes were identified. This scenario may have a high impact on the future management of canine pyoderma caused by *S. coagulans*.

## Figures and Tables

**Table 1:** Antibiotic susceptibility profiles and antibiotic resistance genes detected for the 27 canine pyoderma associated *S. coagulans* isolates studied. Data are only presented for antibiotics with established breakpoints.

Antibiotic	ZD Breakpoint		Number of Isolates (%)			Resistance Determinants (No. Isolates)
	S (mm)	R (mm)	S	I	R	
Penicillin **	≥29 ^a^	≤28 ^a^	10 (37.0%)	-	17 (63.0%)	*blaZ* (16) and/or *mecA* (2)
Oxacillin **	≥18	≤17	25 (92.6%)	-	2 (7.4%)	*mecA* (2)
Enrofloxacin *	≥23	≤16	16 (59.3%)	2 (7.4%)	9 (33.3%)	QRDR mutations: GrlA [S80I, S80R, S80G]; GyrA [S80F, S80Y, E88A, E88G]
Ciprofloxacin **	≥21	≤15	17 (63.0%)	4 (14.8%)	6 (22.2%)	
Moxifloxacin **	≥24	≤20	21 (77.8%)	2 (7.4%)	4 (14.8%)	
Erythromycin **	≥23	≤13	24 (88.9%)	2 (7.4%)	1 (3.7%)	*erm*(B) (1)
Clindamycin *	≥21	≤14	24 (88.9%)	0 (0%)	3 (11.1%)	*erm*(B) (1)
Quinupristin-dalfopristin ***	≥21	<18	27 (100%)	0 (0%)	0 (0%)	-
Tetracycline *	≥23	≤17	26 (96.3%)	0 (0%)	1 (3.7%)	-
Minocycline **	≥19	≤14	27 (100%)	0 (0%)	0 (0%)	-
Tigecycline ***	≥19	<19	27 (100%)	0 (0%)	0 (0%)	
Fusidic acid ***	≥24	<24	25 (92.6%)	-	2 (7.4%)	-
Linezolid **	≥21	≤20	27 (100%)	-	0 (0%)	-
Chloramphenicol **	≥18	≤12	27 (100%)	0 (0%)	0 (0%)	-
Trimethoprim-sulfamethoxazole **	≥16	≤10	27 (100%)	0 (0%)	0 (0%)	-
Rifampicin **	≥20	≤16	27 (100%)	0 (0%)	0 (0%)	-
Gentamicin **	≥15	≤12	27 (100%)	0 (0%)	0 (0%)	-
Amikacin ***	≥18	<18	27 (100%)	-	0 (0%)	-
Tobramycin ***	≥18	<18	27 (100%)	-	0 (0%)	-
Kanamycin ***	≥18	<18	27 (100%)	-	0 (0%)	-

ZD: zone inhibition diameter; S: susceptible; I: intermediate; R: resistant; * Breakpoint established by CLSI for staphylococci isolated from animals, document VET01S ED5 [48]; ** Breakpoint established by CLSI for staphylococci isolated from humans, document M100-S30 [49]; *** Breakpoint established by EUCAST [50]; ^a^ Isolates with a ZD towards penicillin > 29 mm, but with a sharp inhibition border were considered producers of beta-lactamase and thus resistant to penicillin [48].

**Table 2:** Cut-off (CO_WT_) values of *S. coagulans* for six antibiotics that have no breakpoints established by CLSI or EUCAST. The CO_WT_ values were determined based on the distributions of inhibition zone diameters for 27 *S. coagulans* isolates by the NRI method.

	CO_WT_ (mm)	SD (mm)	WT Population (mm)	NWT Population (mm)
Apramycin	18	2.11	≥18	<18
Bacitracin	13	1.85	≥13	<13
Florfenicol	22	2.64	≥22	<22
Mupirocin	32	3.21	≥32	<32
Neomycin	18	2.20	≥18	<18
Novobiocin	30	1.53	≥30	<30

SD: standard deviation; WT: wild-type; NWT: non-wild-type.

**Table 3:** Brief description of the *S. coagulans* (*n* = 27) and *S. schleiferi* (*n* = 1) isolates associated with canine skin infections.

Isolate	Identification	Biological Sample	Year	Laboratory
BIOS-V1 *	*S. coagulans*	skin swab	2015	Lab1
BIOS-V2	*S. coagulans*	skin swab	2012	Lab1
BIOS-V3	*S. coagulans*	skin swab	2014	Lab1
BIOS-V9	*S. coagulans*	skin swab	2018	Lab1
BIOS-V35	*S. coagulans*	skin swab	2018	Lab1
BIOS-V41	*S. coagulans*	perianal skin swab	2018	Lab1
BIOS-V42	*S. coagulans*	skin swab	2001	Lab1
BIOS-V43	*S. coagulans*	skin swab	2004	Lab1
BIOS-V44	*S. coagulans*	skin swab	2005	Lab1
BIOS-V45	*S. coagulans*	perianal fistula swab	2008	Lab1
BIOS-V46	*S. coagulans*	skin swab	1999	Lab1
BIOS-V47	*S. coagulans*	skin swab	2003	Lab1
BIOS-V51	*S. coagulans*	skin swab	2007	Lab1
BIOS-V91	*S. coagulans*	axillar skin swab	2004	Lab1
BIOS-V93	*S. coagulans*	skin swab	2007	Lab1
BIOS-V94	*S. coagulans*	skin swab	2007	Lab1
BIOS-V95	*S. coagulans*	skin swab	2013	Lab1
BIOS-V98	*S. coagulans*	skin swab	2015	Lab1
BIOS-V107	*S. coagulans*	skin swab	2015	Lab1
BIOS-V126 *	*S. coagulans*	skin swab	2016	Lab1
BIOS-V139	*S. schleiferi*	skin swab	2016	Lab1
BIOS-V191	*S. coagulans*	skin swab	2018	Lab2
BIOS-V205	*S. coagulans*	skin swab	2018	Lab2
BIOS-V209	*S. coagulans*	skin swab	2018	Lab2
BIOS-V232	*S. coagulans*	epidermal collarette	2018	Lab2
BIOS-V243	*S. coagulans*	skin swab	2018	Lab2
BIOS-V265	*S. coagulans*	skin swab	2018	Lab2
BIOS-V289	*S. coagulans*	Interdigital skin swab	2018	Lab2

* Isolates collected from the same dog.

## Data Availability

All relevant data have been provided in the paper. Raw data can also be provided by the authors upon reasonable request.

## Supplementary Materials

The following are available online at https://www.mdpi.com/article/10.3390/antibiotics10050518/s1, Table S1: Distribution of inhibition zone diameters of 21 antibiotics for the 27 *S. coagulans* studied; Table S2: Distribution of inhibition zone diameters of six antibiotics with no breakpoint or epidemiological cut-off value for the 27 *S. coagulans* studied; Table S3: Inhibition zone diameters of 27 antibiotics for the single *S. schleiferi* isolate studied; Table S4: Primers used in this study; Table S5: Control strains used in the screening of antibiotic resistance genes.
