# Supplementary material for: Phenotypic and Molecular Traits of Staphylococcus coagulans Associated with Canine Skin Infections in Portugal
Source: Antibiotics (Basel). 2021 May 2;10(5):518. doi: 10.3390/antibiotics10050518 (PMC8147484; doi:10.3390/antibiotics10050518)
Supplement: Supplementary file 1 [file antibiotics-10-00518-s001.zip › Costa_etal_SupplementaryTables._proofs_revised.pdf]

antibiotics

MDPI

Article

Phenotypic and molecular traits of *Staphylococcus coagulans* associated with canine skin infections in Portugal

Sofia Santos Costa[1,*], Valéria Oliveira[1], Maria Serrano[1], Constança Pomba[2,3] and Isabel Couto[1,*]

[1] Global Health and Tropical Medicine (GHTM), Instituto de Higiene e Medicina Tropical (IHMT), Universidade Nova de Lisboa (UNL), Rua da Junqueira 100, 1349-008 Lisboa, Portugal; mmm0005@ihmt.unl.pt (V.O.), a21000870@ihmt.unl.pt (M.S.)
[2] Centre of Interdisciplinary Research in Animal Health (CIISA), Faculty of Veterinary Medicine, University of Lisbon, Avenida da Universidade Técnica, 1300-477, Lisboa, Portugal; cpomba@fmv.ulisboa.pt (C.P.)
[3] GeneVet, Laboratório de Diagnóstico Molecular Veterinário, Rua Quinta da Nora Loja 3B, 2790-140 Carnaxide, Portugal; cpomba@fmv.ulisboa.pt (C.P.)
* Correspondence: scosta@ihmt.unl.pt (S.S.C.); icouto@ihmt.unl.pt (I.C.); Tel.: +351-21-3652652; Fax: +351-21-3632105 (S.S.C. and I.C.)

Table S1. Distribution of inhibition zone diameters of 21 antibiotics for the 27 *S. coagulans* studied.

Antibiotic	Distribution of Inhibition zone diameter (mm)																																							
	6	7	8	9	10	11	12	13	14	15	16	17	18	19	20	21	22	23	24	25	26	27	28	29	30	31	32	33	34	35	36	37	38	39	40	41	42	43	44	45	46
**Penicillin ****																																									
no. isolates	0	0	0	1	0	0	1	0	0	0	0	0	1	1	3	3	1	1	0	0	3	0	0	0	0	1	0	1	0	0	0	0	0	0	0	1	5	1	1	1	1
frequency (%)	0	0	0	3.7	0	0	3.7	0	0	0	0	0	3.7	3.7	11.1	11.1	3.7	3.7	0	0	11.1	0	0	0	0	3.7	0	3.7	0	0	0	0	0	0	0	3.7	18.5	3.7	3.7	3.7	3.7
**Oxacillin ****																																									
no. isolates	0	0	0	0	0	0	0	1	0	1	0	0	0	0	1	7	5	0	1	3	6	1	1	0	0	0	0	0	0	0	0	0	0	0	0	0	0	0	0	0	0
frequency (%)	0	0	0	0	0	0	0	3.7	0	3.7	0	0	0	0	3.7	25.9	18.5	0	3.7	11.1	22.2	3.7	3.7	0	0	0	0	0	0	0	0	0	0	0	0	0	0	0	0	0	0
**Enrofloxacin ***																																									
no. isolates	4	0	1	0	0	0	1	0	2	0	1	1	0	0	1	0	0	3	3	0	0	0	2	0	4	1	2	0	1	0	0	0	0	0	0	0	0	0	0	0	0
frequency (%)	14.8	0	3.7	0	0	0	3.7	0	7.4	0	3.7	3.7	0	0	3.7	0	0	11.1	11.1	0	0	0	7.4	0	14.8	3.7	7.4	0	3.7	0	0	0	0	0	0	0	0	0	0	0	0
**Pradofloxacin**																																									
no. isolates	0	0	0	0	0	0	0	0	0	0	0	1	3	0	0	0	0	1	2	0	1	2	1	0	4	1	6	0	3	2	0	0	0	0	0	0	0	0	0	0	0
frequency (%)	0	0	0	0	0	0	0	0	0	0	0	3.7	11.1	0	0	0	0	3.7	7.4	0	3.7	7.4	3.7	0	14.8	3.7	22.2	0	11.1	7.4	0	0	0	0	0	0	0	0	0	0	0

Cells are colored according to the breakpoints established by respective recommendations; S: green; I: yellow; R: red. * Breakpoint established by CLSI for staphylococci isolated from animals, document VET01S ED5 [1]; ** Breakpoint established by CLSI for staphylococci isolated from humans, document M100-S30 [2]; *** Breakpoint established by EUCAST [3].

Table S1. (Continuation). Distribution of inhibition zone diameters of 21 antibiotics for the 27 *S. coagulans* studied.

Antibiotic	Distribution of Inhibition zone diameter (mm)																																							
	6	7	8	9	10	11	12	13	14	15	16	17	18	19	20	21	22	23	24	25	26	27	28	29	30	31	32	33	34	35	36	37	38	39	40	41	42	43	44	45	46
**Ciprofloxacin ****																																									
no. isolates	4	0	0	0	0	0	0	0	2	0	0	2	1	1	0	0	0	0	0	1	3	2	2	1	0	1	3	1	2	1	0	0	0	0	0	0	0	0	0	0	0
frequency (%)	14.8	0	0	0	0	0	0	0	7.4	0	0	7.4	3.7	3.7	0	0	0	0	0	3.7	11.1	7.4	7.4	3.7	0	3.7	11.1	3.7	7.4	3.7	0	0	0	0	0	0	0	0	0	0	0
**Moxifloxacin ****																																									
no. isolates	0	0	0	0	0	0	0	0	0	0	1	0	0	3	0	1	0	1	0	3	0	1	1	1	6	2	2	0	2	3	0	0	0	0	0	0	0	0	0	0	0
frequency (%)	0	0	0	0	0	0	0	0	0	0	3.7	0	0	11.1	0	3.7	0	3.7	0	11.1	0	3.7	3.7	3.7	22.2	7.4	7.4	0	7.4	11.1	0	0	0	0	0	0	0	0	0	0	0
**Erythromycin ****																																									
no. isolates	1	0	0	0	0	0	0	0	1	0	1	0	0	0	0	0	0	0	0	0	3	1	12	2	4	0	1	0	1	0	0	0	0	0	0	0	0	0	0	0	0
frequency (%)	3.7	0	0	0	0	0	0	0	3.7	0	3.7	0	0	0	0	0	0	0	0	0	11.1	3.7	44.4	7.4	14.8	0	3.7	0	3.7	0	0	0	0	0	0	0	0	0	0	0	0
**Clindamycin ***																																									
no. isolates	1	0	0	0	0	0	0	1	1	0	0	0	0	0	0	0	0	0	1	1	4	1	12	1	3	0	1	0	0	0	0	0	0	0	0	0	0	0	0	0	0
frequency (%)	3.7	0	0	0	0	0	0	3.7	3.7	0	0	0	0	0	0	0	0	0	3.7	3.7	14.8	3.7	44.4	3.7	11.1	0	3.7	0	0	0	0	0	0	0	0	0	0	0	0	0	0
**Quinupristin – dalfopristin *****																																									
no. isolates	0	0	0	0	0	0	0	0	0	0	0	0	0	0	0	0	0	0	0	0	3	5	8	4	6	1	0	0	0	0	0	0	0	0	0	0	0	0	0	0	0
frequency (%)	0	0	0	0	0	0	0	0	0	0	0	0	0	0	0	0	0	0	0	0	11.1	18.5	29.6	14.8	22.2	3.7	0	0	0	0	0	0	0	0	0	0	0	0	0	0	0
**Tetracycline ***																																									
no. isolates	0	0	0	0	0	0	0	0	0	1	0	0	0	0	0	0	0	0	0	0	0	0	2	3	7	4	4	4	0	2	0	0	0	0	0	0	0	0	0	0	0
frequency (%)	0	0	0	0	0	0	0	0	0	3.7	0	0	0	0	0	0	0	0	0	0	0	0	7.4	11.1	25.9	14.8	14.8	14.8	0	7.4	0	0	0	0	0	0	0	0	0	0	0
**Minocycline ****																																									
no. isolates	0	0	0	0	0	0	0	0	0	0	0	0	0	0	0	0	0	0	0	0	1	1	1	4	7	2	5	3	2	0	0	1	0	0	0	0	0	0	0	0	0
frequency (%)	0	0	0	0	0	0	0	0	0	0	0	0	0	0	0	0	0	0	0	0	3.7	3.7	3.7	14.8	25.9	7.4	18.5	11.1	7.4	0	0	3.7	0	0	0	0	0	0	0	0	0
**Tigecycline *****																																									
no. isolates	0	0	0	0	0	0	0	0	0	0	0	0	0	0	0	0	0	0	5	11	5	3	1	0	2	0	0	0	0	0	0	0	0	0	0	0	0	0	0	0	0
frequency (%)	0	0	0	0	0	0	0	0	0	0	0	0	0	0	0	0	0	0	18.5	40.7	18.5	11.1	3.7	0	7.4	0	0	0	0	0	0	0	0	0	0	0	0	0	0	0	0
**Fusidic acid *****																																									
no. isolates	0	0	0	0	0	0	0	0	0	0	0	0	0	0	1	1	0	0	0	0	1	0	0	0	1	2	8	7	2	0	3	1	0	0	0	0	0	0	0	0	0
frequency (%)	0	0	0	0	0	0	0	0	0	0	0	0	0	0	3.7	3.7	0	0	0	0	3.7	0	0	0	3.7	7.4	29.6	25.9	7.4	0	11.1	3.7	0	0	0	0	0	0	0	0	0

Cells are colored according to the breakpoints established by respective recommendations; S: green; I: yellow; R: red. * Breakpoint established by CLSI for staphylococci isolated from animals, document VET01S ED5 [1]; ** Breakpoint established by CLSI for staphylococci isolated from humans, document M100-S30 [2]; *** Breakpoint established by EUCAST [3].

antibiotics MDPI

Table S1. (Continuation). Distribution of inhibition zone diameters of 21 antibiotics for the 27 *S. coagulans* studied.

Antibiotic	Distribution of Inhibition zone diameter (mm)																																							
	6	7	8	9	10	11	12	13	14	15	16	17	18	19	20	21	22	23	24	25	26	27	28	29	30	31	32	33	34	35	36	37	38	39	40	41	42	43	44	45	46
**Linezolid ****																																								
no. isolates	0	0	0	0	0	0	0	0	0	0	0	0	0	0	0	0	0	0	0	0	0	0	1	1	1	6	6	3	5	2	0	0	0	2	0	0	0	0	0	0	0
frequency (%)	0	0	0	0	0	0	0	0	0	0	0	0	0	0	0	0	0	0	0	0	0	0	3.7	3.7	3.7	22.2	22.2	11.1	18.5	7.4	0	0	0	7.4	0	0	0	0	0	0	0
**Chloramphenicol ****																																								
no. isolates	0	0	0	0	0	0	0	0	0	0	0	0	0	0	0	0	0	0	1	1	9	3	7	4	2	0	0	0	0	0	0	0	0	0	0	0	0	0	0	0	0
frequency (%)	0	0	0	0	0	0	0	0	0	0	0	0	0	0	0	0	0	0	3.7	3.7	33.3	11.1	25.9	14.8	7.4	0	0	0	0	0	0	0	0	0	0	0	0	0	0	0	0
**Trimethoprim – sulphamethoxazol ****																																								
no. isolates	0	0	0	0	0	0	0	0	0	0	0	0	0	0	0	0	0	0	0	1	1	8	2	5	7	2	1	0	0	0	0	0	0	0	0	0	0	0	0	0	0
frequency (%)	0	0	0	0	0	0	0	0	0	0	0	0	0	0	0	0	0	0	0	3.7	3.7	29.6	7.4	18.5	25.9	7.4	3.7	0	0	0	0	0	0	0	0	0	0	0	0	0	0
**Rifampicin ****																																								
no. isolates	0	0	0	0	0	0	0	0	0	0	0	0	0	0	0	0	0	0	0	0	0	0	0	0	2	2	5	5	7	3	1	0	1	0	1	0	0	0	0	0	0
frequency (%)	0	0	0	0	0	0	0	0	0	0	0	0	0	0	0	0	0	0	0	0	0	0	0	0	7.4	7.4	18.5	18.5	25.9	11.1	3.7	0	3.7	0	3.7	0	0	0	0	0	0
**Gentamicin ****																																								
no. isolates	0	0	0	0	0	0	0	0	0	0	0	0	1	0	0	0	1	0	0	5	7	8	4	0	1	0	0	0	0	0	0	0	0	0	0	0	0	0	0	0	0
frequency (%)	0	0	0	0	0	0	0	0	0	0	0	0	3.7	0	0	0	3.7	0	0	18.5	25.9	29.6	14.8	0	3.7	0	0	0	0	0	0	0	0	0	0	0	0	0	0	0	0
**Amikacin *****																																								
no. isolates	0	0	0	0	0	0	0	0	0	0	0	0	0	0	0	0	1	0	2	3	8	6	4	0	2	1	0	0	0	0	0	0	0	0	0	0	0	0	0	0	0
frequency (%)	0	0	0	0	0	0	0	0	0	0	0	0	0	0	0	0	3.7	0	7.4	11.1	29.6	22.2	14.8	0	7.4	3.7	0	0	0	0	0	0	0	0	0	0	0	0	0	0	0
**Tobramycin *****																																								
no. isolates	0	0	0	0	0	0	0	0	0	0	0	0	0	0	0	0	1	0	1	3	8	5	8	0	1	0	0	0	0	0	0	0	0	0	0	0	0	0	0	0	0
frequency (%)	0	0	0	0	0	0	0	0	0	0	0	0	0	0	0	0	3.7	0	3.7	11.1	29.6	18.5	29.6	0	3.7	0	0	0	0	0	0	0	0	0	0	0	0	0	0	0	0
**Kanamycin *****																																								
no. isolates	0	0	0	0	0	0	0	0	0	0	0	0	0	0	0	0	0	2	0	3	7	7	7	0	1	0	0	0	0	0	0	0	0	0	0	0	0	0	0	0	0
frequency (%)	0	0	0	0	0	0	0	0	0	0	0	0	0	0	0	0	0	7.4	0	11.1	25.9	25.9	25.9	0	3.7	0	0	0	0	0	0	0	0	0	0	0	0	0	0	0	0

Cells are colored according to the breakpoints established by respective recommendations; S: green; I: yellow; R: red. * Breakpoint established by CLSI for staphylococci isolated from animals, document VET01S ED5 [1]; ** Breakpoint established by CLSI for staphylococci isolated from humans, document M100-S30 [2]; *** Breakpoint established by EUCAST [3].

Table S2. Distribution of inhibition zone diameters of six antibiotics with no breakpoint or epidemiological cut-off value for the 27 *S. coagulans* studied.

Antibiotic	Distribution of Inhibition zone diameter (mm)																																							
	6	7	8	9	10	11	12	13	14	15	16	17	18	19	20	21	22	23	24	25	26	27	28	29	30	31	32	33	34	35	36	37	38	39	40	41	42	43	44	45	46
**Apramycin**																																									
no. isolates	0	0	0	0	0	0	0	0	0	0	0	0	0	0	2	3	4	7	4	6	1	0	0	0	0	0	0	0	0	0	0	0	0	0	0	0	0	0	0	0	0
frequency (%)	0	0	0	0	0	0	0	0	0	0	0	0	0	0	7.4	11.1	14.8	25.9	14.8	22.2	3.7	0	0	0	0	0	0	0	0	0	0	0	0	0	0	0	0	0	0	0	0
**Bacitracin**																																									
no. isolates	0	0	0	0	0	0	0	0	0	0	5	7	4	5	4	2	0	0	0	0	0	0	0	0	0	0	0	0	0	0	0	0	0	0	0	0	0	0	0	0	0
frequency (%)	0	0	0	0	0	0	0	0	0	0	18.5	25.9	14.8	18.5	14.8	7.4	0	0	0	0	0	0	0	0	0	0	0	0	0	0	0	0	0	0	0	0	0	0	0	0	0
**Florfenicol**																																									
no. isolates	0	0	0	0	0	0	0	0	0	0	0	0	0	0	0	0	0	0	0	0	1	1	4	9	8	1	1	0	1	0	1	0	0	0	0	0	0	0	0	0	0
frequency (%)	0	0	0	0	0	0	0	0	0	0	0	0	0	0	0	0	0	0	0	0	3.7	3.7	14.8	33.3	29.6	3.7	3.7	0	3.7	0	3.7	0	0	0	0	0	0	0	0	0	0
**Mupirocin**																																									
no. isolates	0	0	0	0	0	0	0	0	0	0	0	0	0	0	0	0	0	0	0	0	0	0	0	0	0	0	0	0	0	0	3	2	0	5	7	2	4	0	3	0	1
frequency (%)	0	0	0	0	0	0	0	0	0	0	0	0	0	0	0	0	0	0	0	0	0	0	0	0	0	0	0	0	0	0	11.1	7.4	0	18.5	25.9	7.4	14.8	0	11.1	0	3.7
**Neomycin**																																									
no. isolates	0	0	0	0	0	0	0	0	0	0	0	0	0	0	1	0	1	7	7	7	1	1	1	1	0	0	0	0	0	0	0	0	0	0	0	0	0	0	0	0	0
frequency (%)	0	0	0	0	0	0	0	0	0	0	0	0	0	0	3.7	0	3.7	25.9	25.9	25.9	3.7	3.7	3.7	3.7	0	0	0	0	0	0	0	0	0	0	0	0	0	0	0	0	0
**Novobiocin**																																									
no. isolates	0	0	0	0	0	0	0	0	0	0	0	0	0	0	0	0	0	0	0	0	0	0	0	0	0	3	4	3	5	8	4	0	0	0	0	0	0	0	0	0	0
frequency (%)	0	0	0	0	0	0	0	0	0	0	0	0	0	0	0	0	0	0	0	0	0	0	0	0	0	11.1	14.8	11.1	18.5	29.6	14.8	0	0	0	0	0	0	0	0	0	0

Table S3. Inhibition zone diameters of 27 antibiotics for the single *S. schleiferi* isolate studied.

Antibiotic	ZD (mm)	Antibiotic	ZD (mm)	Antibiotic	ZD (mm)	Antibiotic	ZD (mm)
**Penicillin**	11	**Clindamycin**	28	**Chloramphenicol**	28	**Kanamycin**	28
**Oxacillin**	19	**Quinupristin - dalfopristin**	29	**Florfenicol**	30	**Neomycin**	23
**Enrofloxacin**	15	**Tetracycline**	35	**Trimethoprim - sulfamethoxazole**	29	**Apramycin**	25
**Pradofloxacin**	26	**Minocycline**	34	**Rifampicin**	36	**Bacitracin**	20
**Ciprofloxacin**	17	**Tigecycline**	29	**Gentamicin**	25	**Mupirocin**	42
**Moxifloxacin**	25	**Fusidic acid**	17	**Amikacin**	28	**Novobiocin**	36
**Erythromycin**	30	**Linezolid**	34	**Tobramycin**	27		

ZD: inhibition zone diameter.

Table S4. Primers used in this study.

Target Gene	Primers	Nucleotide Sequence (5′-3′)	Amplicon Size (bp)	Reference
***S. coagulans* and *S. schleiferi* identification**				
nuc	nuc-fw	AATGGCTACAATGATAATCACTAA	526	[4]
	nuc-rv	CATATCTGTCTTTCGGCGCG		
**Screening of resistance genes and mutations**				
mecA	mecA_Fw	GGTCCCATTAACTCTGAAG	1040	[5]
	mecA_Rv	AGTTCTGCAGTACCGGATTTGC		
blaZ	blaZ_Fw	GATAAGAGATTTGCCTATGC	533	[6]
	blaZ_Rv	GCATATGTTATTGCTTGACC		
erm(A)	erm(A)_Fw	AAGCGGTAAACCCCTCTGAG	442	[7]
	erm(A)_Rv	TCAAAGCCTGTCGGAATTGG		
erm(B)	erm(B)_Fw	TGGAACAGGTAAAGGGCATT	433	[8]
	erm(B)_Rv	TGTGGTATGGCGGGTAAGTT		
erm(C)	erm(C)_Fw	TCGTAACTGCCATTGAAATA	348	[8]
	erm(C)_Rv	TCACTTTAGGTTTAGGATGAAA		
msr(A)	msr(A)_Fw	GATTGTCCCAAGCCAGTAAA	445	[9]
	msr(A)_Rv	GCCATTTGCACTTTAGGAGA		
mph(C)	mph(C)_Fw	ATGACTCGACATAATGAAAT	900	[10]
	mph(C)_Rv	CTACTCTTTCATACCTAACTC		
vga(A)	vga(A)_Fw	ACCCGAGACATCTTCACCAC	400	[11]
	vga(A)_Rv	GGAAATTGACGAGGGGAGA		
vga(C)	vga(C)_Fw	ACGAATAAAGGGATCGAAGC	510	[9]
	vga(C)_Rv	AGCACATGCACAGGTTTGTA		
fusB	fusB_Fw	ATTCAATCGGAAACCTATAATGATA	292	[12]
	fusB_Rv	TTATATATTTCCGATTTGATGCAAG		
fusC	fusC_Fw	GATATTGATATCTCGGACTT	128	[13]
	fusC_Rv	AGTTGACTTGATGAAGGTAT		
tet(K)	tet(K)_Fw	GTAGCGACAATAGGTAATAGT	361	[14]
	tet(K)_Rv	GTAGTGACAATAAACCTCCTA		
tet(M)	tet(M)_Fw	GTTAAATAGTGTTCTTGGAG	657	[15]
	tet(M)_Rv	CTAAGATATGGCTCTAACAA		
tet(L)	tet(L)_Fw	GTCGGTAATTGGGTTTGTTG	421	This study
	tet(L)_Rv	TGACAGCACGCTAACGATAA		
grlA	grlA_Fw	CAAGAGCGTGCTTTRCCT	300	This study
	grlA_Rv	CTGACTYAATTTCGCTTCAG		
gyrA	gyrA_Fw	ATGAGTGTTATYGTRTCTCGT	261	This study
	gyrA_Rv	CATMGAACCRAAGTTACCTTG		

bp: base pair; **Fw:** "forward"; **Rv:** "reverse"; R: A + G; Y: C + T; M: A + C.

Table S5. Control strains used in the screening of antibiotic resistance genes.

Target Gene	Control strain	Reference	Target Gene	Control strain	Reference
mecA	*S. aureus* SM1	[16]	***vga*(A)**	*S. epidermidis* FMV-51	[18]
blaZ	*S. aureus* SM1	[16]	***vga*(C)**	*S. aureus* 49.1	[11]
***erm*(A)**	*S. aureus* SM39	[8]	***fusB***	*S. epidermidis* FMV-97	[19]
***erm*(B)**	*S. pseudintermedius* 4877/10	[17]	***fusC***	*S. epidermidis* FMV-34	[19]
***erm*(C)**	*S. aureus* SM26	[8]	***tet*(K)**	*S. epidermidis* ATCC12228	[20]
***msr*(A)**	*S. epidermidis* FMV-51	[18]	***tet*(M)**	*S. aureus* H4/09	[11]
***mph*(C)**	*S. epidermidis* FMV-51	[18]	***tet*(L)**	*S. aureus* 25.1	[11]

References

1. Clinical and Laboratory Standards Institute (CLSI). Performance standards for antimicrobial disk and dilution susceptibility tests for bacteria isolated from animals, 5th Edition. CLSI Supplement VET01S. USA, 2020.
2. Clinical and Laboratory Standards Institute (CLSI). Performance standards for antimicrobial susceptibility testing, 30th Edition. CLSI Supplement M100. USA, 2020.
3. European Committee on Antimicrobial Susceptibility Testing (EUCAST). Breakpoint tables for interpretation of MICs and zone diameters, v11.0. 2021. http://www.eucast.org
4. Sasaki, T., Tsubakishita, S., Tanaka, Y., Sakusabe, A., Ohtsuka, M., Hirotaki, S., Kawakami, T., Fukata, T., Hiramatsu, K. Multiplex-PCR method for species identification of coagulase-positive staphylococci. *J Clin Microbiol* **2010**, *48*, 765-769. doi: 10.1128/JCM.01232-09.
5. Petinaki, E.; Arvaniti, A.; Dimitracopoulos, G.; Spiliopoulou, I. Detection of *mecA*, *mecR1* and *mecI* genes among clinical isolates of methicillin-resistant staphylococci by combined polymerase chain reactions. *J Antimicrob Chemother* **2001**, *47*, 297-304. doi: 10.1093/jac/47.3.297
6. Milheiriço, C.; Portelinha, A.; Krippahl, L.; de Lencastre, H.; Oliveira, D.C. Evidence for a purifying selection acting on the β-lactamase locus in epidemic clones of methicillin-resistant *Staphylococcus aureus*. *BMC Microbiol* **2011**, *11*, 76. doi: 10.1186/1471-2180-11-76
7. Jensen, L.B.; Hammerum, A.M.; Bager, F.; Aarestrup, F.M. Streptogramin resistance among *Enterococcus faecium* isolated from production animals in Denmark in 1997. *Microb Drug Resist* **2002**, *8*, 369-374. doi: 10.1089/10766290260469642
8. Costa, S.S.; Palma, C.; Kladec, K.; Fessler, A.T.; Viveiros, M.; Melo-Cristino, J.; Schwarz, S.; Couto, I. Plasmid-borne antimicrobial resistance of *Staphylococcus aureus* isolated in a hospital in Lisbon, Portugal. *Microb Drug Resist* **2016**, *22*, 617-626. doi: 10.1089/mdr.2015.0352
9. Ferreira, C.; Costa, S.S.; Serrano, M.; Oliveira, K.; Trigueiro, G.; Pomba, C.; Couto, I. Clonal Lineages, Antimicrobial Resistance, and PVL Carriage of *Staphylococcus aureus* associated to skin and soft-tissue infections from ambulatory patients in Portugal. *Antibiotics* **2021**, *10*, 345. https://doi.org/10.3390/antibiotics10040345
10. Schnellmann, C.; Gerber, V.; Rossano, A.; Jaquier, V.; Panchaud, Y.; Doherr, M.G.; Thomann, A.; Straub, R.; Perreten, V. Presence of new *mecA* and *mph*(C) variants conferring antibiotic resistance in *Staphylococcus* spp. isolated from the skin of horses before and after clinic admission. *J Clin Microbiol* **2006**, *44*, 4444-4454. doi: 10.1128/JCM.00868-06
11. Couto, N.; Belas, A.; Kadlec, K.; Schwarz, S.; Pomba, C. Clonal diversity, virulence patterns and antimicrobial and biocide susceptibility among human, animal and environmental MRSA in Portugal. *J Antimicrob Chemother* **2015**, *71*, 1479-1487. doi: 10.1093/jac/dkv141
12. O'Neill, A.J.; Larsen, A.R.; Henriksen, A.S.; Chopra, I. A fusidic acid-resistant epidemic strain of *Staphylococcus aureus* carries the *fusB* determinant, whereas *fusA* mutations are prevalent in other resistant isolates. *Antimicrob Agents Chemother* **2004**, *48*, 3594-3597. doi: 10.1128/AAC.48.9.3594-3597.2004
13. Castanheira, M.; Watters, A.A.; Bell, J.M.; Turnidge, J.; Jones, R.N. Fusidic acid resistance rates and prevalence of resistance mechanisms among *Staphylococcus spp*. isolated in North America and Australia, 2007-2008. *Antimicrob. Agents Chemother* **2010**, *54*, 3614-3617. doi: 10.1128/AAC.01390-09
14. Strommenger, B., Kettlitz, C., Werner, G., Witte, W. Multiplex PCR assay for simultaneous detection of nine clinically relevant antibiotic resistance genes in *Staphylococcus aureus*. *J Clin Microbiol* **2003**, *41*, 4089-4094. doi: 10.1128/jcm.41.9.4089-4094.2003.
15. Aarestrup, F., Agerso, Y., Gerner–Smidt, P., Madsen, M., Jensen, L. Comparison of antimicrobial resistance phenotypes and resistance genes in *Enterococcus faecalis* and *Enterococcus faecium* from humans in the community, broilers, and pigs in Denmark. *Diagn Microbiol Infect Dis* **2000**, *37*, 127-137. doi: 10.1016/s0732-8893(00)00130-9.

16. Costa, S.S., Falcão, C., Viveiros, M., Machado, D., Martins, M., Melo-Cristino, J., Amaral, L., Couto, I. Exploring the contribution of efflux on the resistance to fluoroquinolones in clinical isolates of *Staphylococcus aureus*. BMC Microbiology. 2011;11:241. doi: 10.1186/1471-2180-11-241
17. Couto, N., Monchique, C., Belas, A., Marques, C., Gama, L.T., Pomba, C. Trends and molecular mechanisms of antimicrobial resistance in clinical staphylococci isolated from companion animals over a 16 year period. *J Antimicrob Chemother* **2016,** *71*, 1479-1487. doi: 10.1093/jac/dkw029.
18. Holtreman, F. D. Characterization of plasmids of *Staphylococcus epidermidis* and correlation with efflux-mediated resistance. 2018. MSc Thesis in Biomedical Sciences, Universidade NOVA de Lisboa. http://hdl.handle.net/10362/53495
19. Rosa, M.S.R. O. Contribution of efflux to antimicrobial resistance in *Staphylococcus epidermidis*. 2017. MSc Thesis in Biomedical Sciences, Universidade NOVA de Lisboa. http://hdl.handle.net/10362/20443
20. MacLea, K., Trachtenberg, A. Complete genome sequence of *Staphylococcus epidermidis* ATCC 12228 chromosome and plasmids, generated by long-read sequencing. *Genome Announc*. 2017;5: e00954-17. doi: 10.1128/genomeA.00954-17
